# Pathology Associated with AAV Mediated Expression of Beta Amyloid or C100 in Adult Mouse Hippocampus and Cerebellum

**DOI:** 10.1371/journal.pone.0059166

**Published:** 2013-03-13

**Authors:** Eleanor S. Drummond, Jill Muhling, Ralph N. Martins, Linda K. Wijaya, Erich M. Ehlert, Alan R. Harvey

**Affiliations:** 1 School of Anatomy, Physiology and Human Biology, The University of Western Australia, Western Australia, Australia; 2 School of Psychiatry and Clinical Neurosciences, The University of Western Australia, Western Australia, Australia; 3 Centre of Excellence for Alzheimer’s Disease Research and Care, School of Exercise, Biomedical and Health Sciences, Edith Cowan University, Western Australia, Australia; 4 Laboratory for Neuroregeneration, Netherlands Institute for Neuroscience, Royal Academy of Arts and Sciences, Amsterdam, The Netherlands; Case Western Reserve University, United States of America

## Abstract

Accumulation of beta amyloid (Aβ) in the brain is a primary feature of Alzheimer’s disease (AD) but the exact molecular mechanisms by which Aβ exerts its toxic actions are not yet entirely clear. We documented pathological changes 3 and 6 months after localised injection of recombinant, bi-*cis*tronic adeno-associated viral vectors (rAAV2) expressing human Aβ40-GFP, Aβ42-GFP, C100-GFP or C100^V717F^-GFP into the hippocampus and cerebellum of 8 week old male mice. Injection of all rAAV2 vectors resulted in wide-spread transduction within the hippocampus and cerebellum, as shown by expression of transgene mRNA and GFP protein. Despite the lack of accumulation of Aβ protein after injection with AAV vectors, injection of rAAV2-Aβ42-GFP and rAAV2- C100^V717F^-GFP into the hippocampus resulted in significantly increased microgliosis and altered permeability of the blood brain barrier, the latter revealed by high levels of immunoglobulin G (IgG) around the injection site and the presence of IgG positive cells. In comparison, injection of rAAV2-Aβ40-GFP and rAAV2-C100-GFP into the hippocampus resulted in substantially less neuropathology. Injection of rAAV2 vectors into the cerebellum resulted in similar types of pathological changes, but to a lesser degree. The use of viral vectors to express different types of Aβ and C100 is a powerful technique with which to examine the direct *in vivo* consequences of Aβ expression in different regions of the mature nervous system and will allow experimentation and analysis of pathological AD-like changes in a broader range of species other than mouse.

## Introduction

There is a large body of evidence suggesting that beta amyloid (Aβ) accumulation in the brain may be a primary cause of Alzheimer’s disease (AD). However, exactly how Aβ contributes to AD pathology and the mechanisms involved are still not yet fully understood. The two main isoforms of Aβ are Aβ40 and Aβ42. While it is widely accepted that Aβ42 is more neurotoxic than Aβ40, surprisingly few studies have attempted to determine the individual physiological and pathological effects directly attributed to Aβ40 and Aβ42 *in vivo* and even fewer studies have directly compared the effects of Aβ40 and Aβ42 expression [1], [2]. It is important to understand the physiological and pathological functions of each Aβ isoform as many proposed therapies for AD act by modulating Aβ levels [3], [4], [5], [6].

It is difficult to determine the exact role of Aβ in AD using the current available animal models, which are predominantly transgenic mice. Therefore it was the aim of this study to develop an alternative animal model for AD research using viral vectors to initiate localised expression of human Aβ. There are many benefits associated with using viral vectors to develop animal models of disease. For example, viral vectors can rapidly express transgene proteins localised to desired brain regions, or even specific cell types. Viral vectors can also induce transgene expression in many species and at specific ages, hence preventing developmental or other unwanted compensatory variables in response to life-long transgene expression. Viral vectors also allow for the expression of multiple genes with much greater ease than in transgenic mice, a feature that is particularly important when studying a multi-factorial disease such as AD.

To date, two studies have used viral vectors to directly express Aβ. The first study developed viral vectors that produced Aβ40-BRI or Aβ42-BRI fusion proteins [2]. The BRI protein is involved in amyloid deposition in Familial British Dementia and Familial Danish Dementia, and fusion of BRI to Aβ results in enhanced secretion of Aβ-BRI fusion proteins. Expression of Aβ42-BRI resulted in development of amyloid plaques at 3 months post-injection and injection of a combination of BRI-Aβ42 and BRI-Aβ40 vectors resulted in cognitive impairment. The second study developed a LV-Aβ42 vector, which was injected into the primary motor cortex of rats [7]. While resulting brain pathology was only examined at the early post-injection time point of 4 weeks, Aβ42 expression resulted in astrogliosis, increased TNF-α expression and increased tau phosphorylation.

In the present study, recombinant bi-*cis*tronic adeno-associated viral vectors (rAAV2) were constructed, expressing either human Aβ40, Aβ42, C100 or C100^V717F^. Direct expression of human Aβ40 and Aβ42 was performed in an attempt to determine the individual functions of each of these forms of Aβ, while bypassing the possible confounding functional effects of other APP fragments such as sAPPα, N-APP, C100 and AICD, which are also produced during APP processing [8], [9], [10], [11], [12]. Viral vectors expressing C100, the immediate precursor to Aβ, with and without the Indiana mutation (V717F) were also developed in case Aβ expression and/or functional effects were influenced by production from a precursor protein. The Indiana mutation results in preferential production of Aβ42 rather than Aβ40 [13], [14], so this strategy allowed for the indirect comparison of Aβ40 and Aβ42 by comparing the downstream effects of C100 and C100^V717F^ expression. Each of these rAAV2 vectors was injected into the adult mouse hippocampus and cerebellum, brain regions that are vulnerable or relatively spared in AD respectively. rAAV2-GFP and PBS injections served as controls. A targeted number of changes associated with increased Aβ were examined, including glial reactivity (both astrogliosis and microgliosis), neuronal degeneration, blood brain barrier disruption and expression of apolipoprotein E (apoE), clusterin, synaptophysin and presenilin 1 (PS1).

## Materials and Methods

### Ethics Statement

All animal experimentation was approved by the UWA Animal Ethics Committee and conformed to published NHMRC guidelines.

### Plasmid and rAAV2 Construction

rAAV2 vectors were generated from the pTRUF12 plasmid, which contains two inverted terminal repeat sequences from AAV serotype 2, the inserted transgene under the control of the cytomegalovirus early enhancer-chicken beta actin (CMV-CAG) promoter and an additional intron sequence with an enhancer element. Four novel plasmids were generated containing transgene sequences for Aβ40, Aβ42, C100 and C100^V717F^. The pTRUF12 plasmid without an inserted transgene was used as the GFP control plasmid. An internal ribosome entry site (IRES) allowed the bi-*cis*tronic expression of the inserted transgene with enhanced GFP (eGFP), resulting in transgene and GFP production as individual proteins within each transduced cell. All plasmids were sequenced to ensure that the transgene sequences were in the correct orientation and that no unwanted mutations were present.

rAAV2 vectors were produced using a previously described method [15]. An infection assay of HEK 293T cells was performed to ensure infectivity and titres were determined by quantitative PCR (qPCR) for viral genomic copies (GC) extracted from DNase-treated viral particles. Titres ranged from 1.4×10^12^ to 4.1×10^12^ GC/ml.

### In vitro Transfection

HEK 293T, PC12 (A.T.C.C. CRL-1721) and HeLa cells were grown to approximately 90% confluency. HEK 293T and HeLa cells were maintained in DMEM media (Sigma) containing 10% fetal calf serum, 2 mM L-glutamine (Invitrogen), 100 Units/ml penicillin (Invitrogen) and 100 µg/ml streptomycin (Invitrogen). PC12 cells were maintained in DMEM media containing 5% fetal calf serum, 10% horse serum, 2 mM L-glutamine, 100 Units/ml penicillin, 100 µg/ml streptomycin and 0.1 mM non-essential amino acids (Invitrogen). All cell types were transfected using 4 µg of plasmid DNA and 10 µl of Lipofectamine 2000 (Invitrogen) according to the manufacturer’s instructions. Cells were examined for GFP expression 24 and 48 hrs after transfection. Transfection experiments were performed a minimum of three times for each plasmid construct.

Media and cell lysates were collected for Western blot 48 hours post-transfection. Cells were lysed with RIPA buffer (150 mM NaCl, 50 mM Tris-HCl, 1% Triton-X100, 0.5% sodium deoxycholate, 0.1% SDS, 0.1 mM PMSF). Cell media was immunoprecipitated for Aβ using 6 E10 antibody (Signet) and GammaBind Plus Sepharose beads (Amersham/GE Healthcare). Western blot was used to assay GFP, C100 and Aβ levels in cell homogenates and immunoprecipitated media. Briefly, between 25–200 µg of cell homogenate and immunoprecipitated media was added to SDS loading buffer (166 mM Tris-HCl, 8% sodium dodecylsulfate, 4% glycine, 2.5% 2-mercaptoethanol, pinch of phenol red, pH 6.8), boiled at 95°C for 10 min and loaded onto tris-trycine polyacrylamide gels. Proteins were separated using electrophoresis and transferred to nitrocellulose membranes at 250 mA overnight at 4°C. Membranes were blocked with 5% skim milk in TBS for 1 hr at room temperature and immunoblotted for GFP (1∶3,000; Sigma) and C100/Aβ (WO2; 1∶2,500; kindly provided by Professor Colin Masters at University of Melbourne, Vic, Australia). Membranes were also blotted for β-actin (1∶20,000; Abcam) to ensure consistent protein loading. Protein bands were detected using enhanced chemiluminescence (ECL; Amersham Biosciences) and developed onto film. 50 µg of brain protein homogenate from APP^SWE^ transgenic mouse brain was used as a positive control for Aβ detection.

### Surgery and Tissue Processing

Eight week old male C57Bl6/J mice were anesthetised by an intraperitoneal (i.p.) injection (10 µl/g) of a 1∶1 mixture of ketamine (100 mg/ml) and xylazine (20 mg/ml) that was diluted 1∶10 in sterile saline. Mice were secured in a stereotaxic frame and a small section of the skull was removed using a dental drill at the co-ordinates of 1.9 mm rostral and 1.3 mm lateral relative to lambda. For cerebellar injections a burr hole was drilled at approximately 1 mm caudal to lambda and 2 mm lateral to the midline. 1 µl of rAAV2 vector (10^12^ GC/ml) or vehicle (phosphate buffered saline; PBS, pH 7.4) was directly injected into the hippocampus (1.5 mm ventral from the dura) and cerebellum using a pulled glass capillary at a rate of 0.2 µl/min. After each injection, the glass capillary was left in place for an additional 2 minutes before withdrawal. The skull fragment was then replaced and the skin closed using 4.0 sutures (Ethilon; Johnson & Johnson, Australia). After each experimental procedure mice received a subcutaneous injection of buprenorphine (0.02 mg/kg; Temgesic; Reckitt & Colman, Hull, UK) and an intramuscular injection of Benacillin (25 µl, Troy Laboratories Pty. Ltd. Australia).

The number of animals included in each group and the type of molecular analysis carried out at 3 months post-injection is shown in Table 1. Analysis of cerebellar rAAV2-C100-GFP injections was not possible due to problems with injection and thus consistent transduction of neurons in the cerebellar cortex. rAAV2-GFP, rAAV2-C100^V717F^-GFP and rAAV2-Aβ42-GFP were used as representative groups for further analysis at 6 months post-injection.

**Table 1:** Animal experimental groups used for different analysis techniques.

Vector injected	n	Post-injection time	Analysis techniques
AAV2-C100-GFP	4	3 months	Immunohistochemistry/PCR
AAV2-C100^V717F^-GFP	8	3 months	Immunohistochemistry/PCR
AAV2- Aβ40-GFP	4	3 months	Immunohistochemistry/PCR
AAV2- Aβ42-GFP	4	3 months	Immunohistochemistry/PCR
AAV2-GFP	7	3 months	Immunohistochemistry/PCR
PBS	4	3 months	Immunohistochemistry/PCR
AAV2-C100^V717F^-GFP	4	3 months	Western blot
AAV2- Aβ42-GFP	4	3 months	Western blot
AAV2-GFP	4	3 months	Western blot
AAV2-C100^V717F^-GFP	4	6 months	Immunohistochemistry/PCR
AAV2- Aβ42-GFP	4	6 months	Immunohistochemistry/PCR
AAV2-GFP	4	6 months	Immunohistochemistry/PCR

For cryosectioning, mice received an overdose of sodium pentobarbitone (i.p.; Lethabarb, Virbac) and were transcardially perfused with PBS (pH 7.4) containing 0.1% heparin followed by 4% paraformaldehyde in 0.1 M phosphate buffer (pH 7.4). Brains were post-fixed in 4% paraformaldehyde for 2 hours, cryoprotected by sinking in 30% sucrose and incubated in increasing concentrations of Jung freezing medium, all at 4°C. Brains were frozen in 100% Jung Freezing medium by immersion into isopentane on a bed of dry ice and stored at -80°C until sectioning. Serial coronal cryosections were cut at a thickness of 25 µm for slides 1–13 of each series (for immunohistochemistry) and a thickness of 14 µm for slides 14–15 of each series (for PCR), resulting in each slide containing between 5–6 serial sections, which were approximately 350 µm apart. Every section through the hippocampus and cerebellum was collected.

For Western blot and ELISA, mice received an overdose of sodium pentobarbitone (i.p.). The injected hippocampus, contralateral hippocampus, cerebellum and rest of the brain were collected individually, snap frozen in liquid nitrogen and stored at −80°C until homogenisation. Brain samples were homogenised in a volume of ice-cold PBS containing protease inhibitor (Roche) three times the weight of the tissue sample. Total protein was quantified using the Micro BCA protein assay kit (Pierce) and samples were stored at −80°C.

### Immunohistochemistry and Density Quantification

Primary antibodies used for immunohistochemistry were rabbit anti-GFP (Millipore; 1∶400), rabbit anti-GFAP (Sigma; 1∶500), mouse anti-NeuN (Chemicon; 1∶500), rabbit anti-IBA-1 (Wako; 1∶1000), rabbit anti-MAP2 (Millipore; 1∶500) and rabbit CT20 (Calbiochem; 1∶16,000). Secondary antibodies used were anti-rabbit Alexa 488 (Invitrogen; 1∶400), anti-mouse Cy3 (Jackson; 1∶300) and anti-rabbit Cy3 (Jackson; 1∶300).

For immunohistochemistry, cryosections were washed with PBS, blocked for 1 hr in blocking buffer (PBS; 10% normal goat serum; 0.2% Triton-X100) and incubated overnight (4°C) with primary antibodies diluted in blocking buffer. Sections were washed with PBS, incubated with secondary antibodies diluted in blocking buffer for 2 hrs at room temperature, counterstained with Hoechst 33342 (Sigma; 4 µl/ml in PBS) and coverslipped with Dako fluorescent mounting media. Images were collected using a multiphoton laser scanning confocal microscope.

Immunoreactivity for GFAP, IBA-1 and MAP2 was semi-quantified as staining density in the injected hippocampus and cerebellum. Low magnification confocal images were collected of the injected and contralateral hippocampi and cerebellar hemispheres. Three serial sections per brain in each brain region were analysed, the central section containing the injection site to maintain consistency between animals. Images were collected using the lowest magnification that allowed for adequate visualisation of immuno-positive staining. Thus, GFAP images were collected at 5×magnification, IBA-1 images were collected at 10×magnification and MAP2 images were collected at 20×magnification. Because of the higher magnification necessary to image MAP2 staining, images of the hippocampus were collected from both the dentate gyrus and CA3 to ensure that the entire transduced region was imaged. Every image for each immunohistochemistry stain was collected using the same imaging conditions.

GFAP, IBA-1 and MAP2 staining density was quantified using ImageJ analysis software (NIH). The area for quantification of hippocampal staining was defined by the hippocampal anatomical boundaries and density was measured in the injected and contralateral hippocampi. In the cerebellum the analysis region was defined by a rectangular area containing the GFP positive transduced region in the injected hemisphere and the corresponding region of the same area in the contralateral hemisphere. The staining density of the injected hippocampus and cerebellar hemisphere was calculated as a percentage of the corresponding contralateral hippocampus or cerebellar hemisphere and averaged across three sections per brain region. While it is acknowledged that injection of rAAV2 vectors could cause distal changes in contralateral brain regions, which would not be accounted for using this quantification method, the staining density was calculated this way in an attempt to standardise staining intensity across multiple immunohistochemistry runs. Furthermore, general observation of immunoreactivity in the contralateral hemisphere showed minimal distal effects and no individual differences between rAAV2 vectors and vehicle control, suggesting that contralateral differences would not bias results.

### Aβ Immunohistochemistry

Multiple antibodies against Aβ were trialled including; anti-mouse 6E10 (Signet), anti-rabbit pan Aβ (Zymed), anti-mouse 4G8 (Signet) and biotinylated Aβ40 and Aβ42 antibodies (gifts from Dr. Pankaj Mehta, NYS Institute for Basic Research, New York, USA). Extensive optimisation of the immunohistochemistry protocol was also performed, trialling heat and formic acid antigen retrieval methods either alone or in combination, multiple blocking reagents including 10% goat serum, 20% fetal bovine serum, 0.2% bovine serum albumin and mouse-on-mouse blocking reagent (M.O.M; Vector Laboratories) and both fluorescent and peroxidase immunohistochemistry. Positive control tissue sections from 18 month old APP^SWE^ transgenic mice were used in every Aβ immunohistochemistry trial.

### IgG Staining and Quantification

Mouse IgG was stained by incubation with anti-mouse secondary antibodies. Briefly, sections were washed with PBS, blocked for 1 hr in blocking buffer (PBS; 10% normal goat serum; 0.2% Triton-X100) and incubated with anti-mouse secondary antibodies diluted in blocking buffer for 2 hrs at room temperature (anti-mouse Cy3; 1∶300; Jackson or anti-mouse FITC; 1∶100; MP Biochem). Sections were counterstained with Hoechst 33342 (Sigma; 4 µl/ml in PBS) and coverslipped with Dako fluorescent mounting media. Mouse IgG positive cells were identified by their distinctive morphology of intense circular IgG staining closely surrounding a nucleus. All mouse IgG positive cells were counted in three serial sections per brain in the hippocampus and the cerebellum, with the injection site as the central section for each brain region.

For semi-quantitative analysis of IgG staining, we used sections immunostained for NeuN followed by a Cy3 anti-mouse secondary antibody. This allowed for consistent intensity grading of NeuN immunoreactivity versus non-specific IgG staining by comparing label in the injected hemisphere with the contralateral hemisphere. IgG staining intensity was graded in all serial sections through the hippocampus and cerebellum on a scale of 0–3, based on the relative intensity of extracellular IgG staining to positive staining in the surrounding densely packed neuronal layers (including the dentate gyrus, the pyramidal cell layer of CA regions and the granule cell layer in the cerebellum). Grading definitions: 0; no excess IgG staining, 1; stronger IgG staining intensity in neuronal layers than in the surrounding extracellular space, 2; same IgG staining intensity in the neuronal layers and extracellular space, 3; stronger IgG staining intensity in the extracellular space than in neuronal layers.

### Western Blot

Total levels of C100, Aβ and GFP protein were measured using the Western blot described above. 200 µg of total protein homogenate from each brain region was loaded and proteins were detected using the following primary antibodies; GFP (1∶3,000; Sigma), C100/Aβ (6E10; 1∶1,000; Signet) and β-actin (1∶20,000; Abcam).

### Aβ ELISA

Aβ40 and Aβ42 levels were measured in the injected hippocampus and cerebellum after injection of rAAV2-GFP, rAAV2-C100^V717F^-GFP or rAAV2-Aβ42-GFP. Protein was extracted from tissue samples using a previously published method designed to maximise the amount of Aβ detection using ELISA [16]. Aβ40 and Aβ42 levels were quantified using the INNO-BIA plasma Aβ forms test (Innogenetics) according to manufacturer’s instructions. This kit combines ELISA and multiplex technology to assay Aβ40 and Aβ42 levels in the same sample, allowing high sensitivity detection of Aβ using a small volume of sample. All samples were assayed in triplicate. A small amount of non-specific background protein detection was observed in the Aβ42 assay results. Therefore, we conservatively deemed that any values that were greater than two standard deviations outside this background level to contain significant levels of Aβ42.

### PCR

RNA from the injected hippocampus, contralateral hippocampus and transduced cerebellar hemisphere was extracted from cryosections adjacent to those used for immunohistochemistry. Tissue was collected from five serial sections in the hippocampus and three serial sections in the cerebellum, with the injection site as the central section in each brain region. Injected and contralateral hippocampi and the transduced cerebellar hemisphere were carefully removed from whole brain sections using a scalpel blade and were collected individually. RNA was extracted from tissue samples using the FFPE RNeasy miniprep kit (Qiagen) according to the manufacturer’s instructions and nucleic acid quality was assessed using a Nanodrop spectrophotometer. Between 300 ng and 400 ng of RNA was used in reverse transcription reactions performed using the Quantitect RT kit, which included a genomic DNA removal step (Qiagen).

Each qPCR reaction was performed using IQ PCR mix (BioRad) with 2 µl cDNA template, 5 µM of each primer and 1 µl of sterile water to a final volume of 10 µl, using a RotorGene RG 6000 PCR machine. Primer sequences and specific qPCR conditions are shown in Table 2. All runs were repeated, and values for each sample averaged. All PCRs were initially validated by agarose gel electrophoresis to check product specificity and size, with correct single bands excised from gels and purified using a Gel Purification kit (Qiagen) and sequenced using Big Dye Terminator v3.1 mix (Applied Biosystems) to confirm product identity. L19 and PPIA housekeeping genes were used for qPCR analysis and it was ensured that normalisation to each of these housekeeping genes resulted in similar expression patterns.

**Table 2:** Primers and PCR conditions.

Primer	Sequence (5`-3`)	Annealing temp (^o^C)	GenBank ID
L19 F *	CTGAAGGTCAAAGGGAATGTG	51	NM_031103
L19 R *	GGACAGAGTCTTGATGATCTC		
PPIA F *	AGCATACAGGTCCTGGCATC	62	BC059141
PPIA R *	TTCACCTTCCCAAAGACCAC		
Transgene F	TTGCAGAAGATGTGGGTTCA	59	N/A
Transgene R	GGTACCGCAATACCGGAGTA		
Synaptophysin F	CTGGCCACCTACATCTTCCT	58	NM_009305
Synaptophysin R	CCACATGAAAGCGAACACTG		
Clusterin F	TATGCACGTGTCTGCAGGAG	58	NM_013492
Clusterin R	CGCCGTTCATCCAGAAGTAG		
ApoE F	AACCGCTTCTGGGATTACCT	58	NM_009696.3
ApoE R	AGCTGTTCCTCCAGCTCCTT		
PS-1 F	CACCCCATTCACAGAAGACA	58	NM_008943
PS-1 R	TAGTCCACGGCGACATTGTA		

* denotes reference gene.

### Statistics

All statistics were performed using SPSS (Version 17). Two-tailed independent t-tests were initially used to determine if there was a significant difference between PBS and AAV2-GFP control groups in each brain region for all measures. The Levene’s test for equality of variances was also performed to ensure normality of the distribution and to test the equality of variances assumption. As there was no difference observed between brain regions injected with PBS and rAAV2-GFP for any measure, rAAV2-GFP was used as the only control group for all further analysis, as this was the most appropriate control for comparison with experimental rAAV2 vectors. Univariate, one-way ANOVA was used when comparing more than two groups and the Bonferroni post-hoc test was used to determine individual significant differences between groups. The non-parametric Kruskal-Wallis Test was used to perform statistics on the intensity grading results from IgG staining. In all cases p<0.05 was deemed significant.

## Results

### In vitro Production of C100 and Aβ

**Figure 1:**
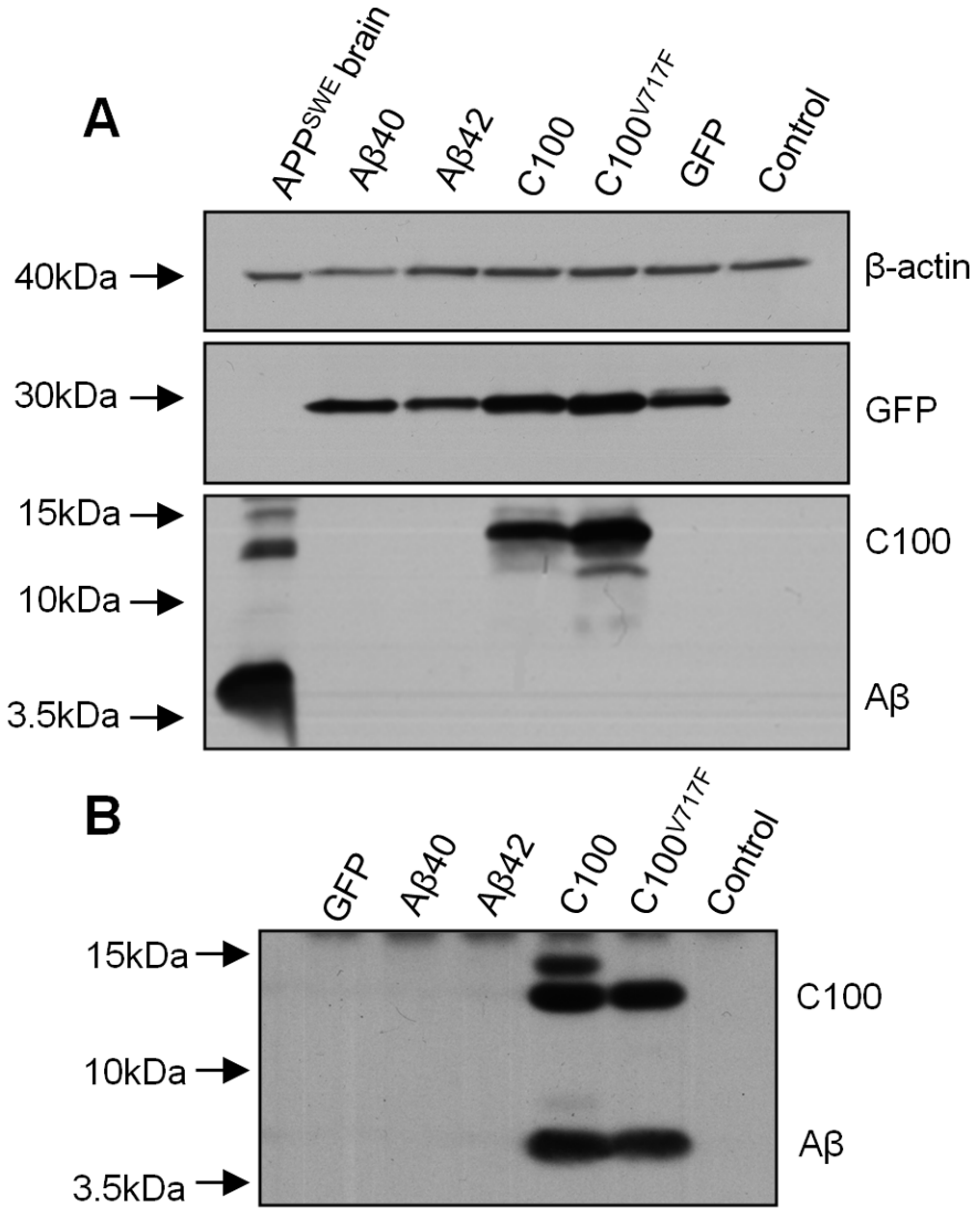
C100 and Aβ protein production *in vitro* after plasmid transfection. Analysis of C100 and Aβ protein production *in vitro* after transfection with plasmids expressing Aβ40-GFP, Aβ42-GFP, C100-GFP or C100^V717F^-GFP in cell homogenates (**A**) and media (**B**). C100 protein was only detected in cell homogenates after transfection with C100-GFP and C100^V717F^-GFP plasmids. No Aβ was detected in cell homogenates after transfection with any plasmid. APP^SWE^ brain homogenate was used as a positive control for Aβ detection. Strong GFP protein expression indicated successful transfection using all plasmids and β-actin was used as a loading control. Immunoprecipitation of Aβ from the cell culture media revealed Aβ in the media from cells transfected with C100-GFP and C100^V717F^-GFP plasmids. Control refers to non-transfected cells.

To confirm that rAAV2 transgenes were translated into protein, HEK 293T, PC12 and HeLa cells were transfected with C100-GFP, C100^V717F^-GFP, Aβ40-GFP and Aβ42-GFP plasmids. Strong GFP protein expression was observed using microscopy and Western blot (Figure 1A), indicating that transfection with all plasmids was successful in each cell type. Transfection with C100-GFP and C100^V717F^-GFP plasmids resulted in abundant intracellular C100 protein expression (Figure 1A). Immunohistochemistry using the 6E10 antibody confirmed that C100 protein was only present in GFP-positive transfected cells, with immuno-positive staining observed throughout the cytoplasm in a punctate pattern (data not shown).

No intracellular Aβ protein could be detected using Western blot (Figure 1A) or immunohistochemistry after transfection with all plasmids, in any cell type. Immunoprecipitation of Aβ from the media of transfected cells showed that Aβ was secreted from cells transfected with C100-GFP and C100^V717F^-GFP plasmids, but we did not detect any secreted Aβ from cells transfected with Aβ40-GFP and Aβ42-GFP plasmids (Figure 1B).

### In vivo Transduction of Hippocampus and Cerebellum

**Figure 2:**
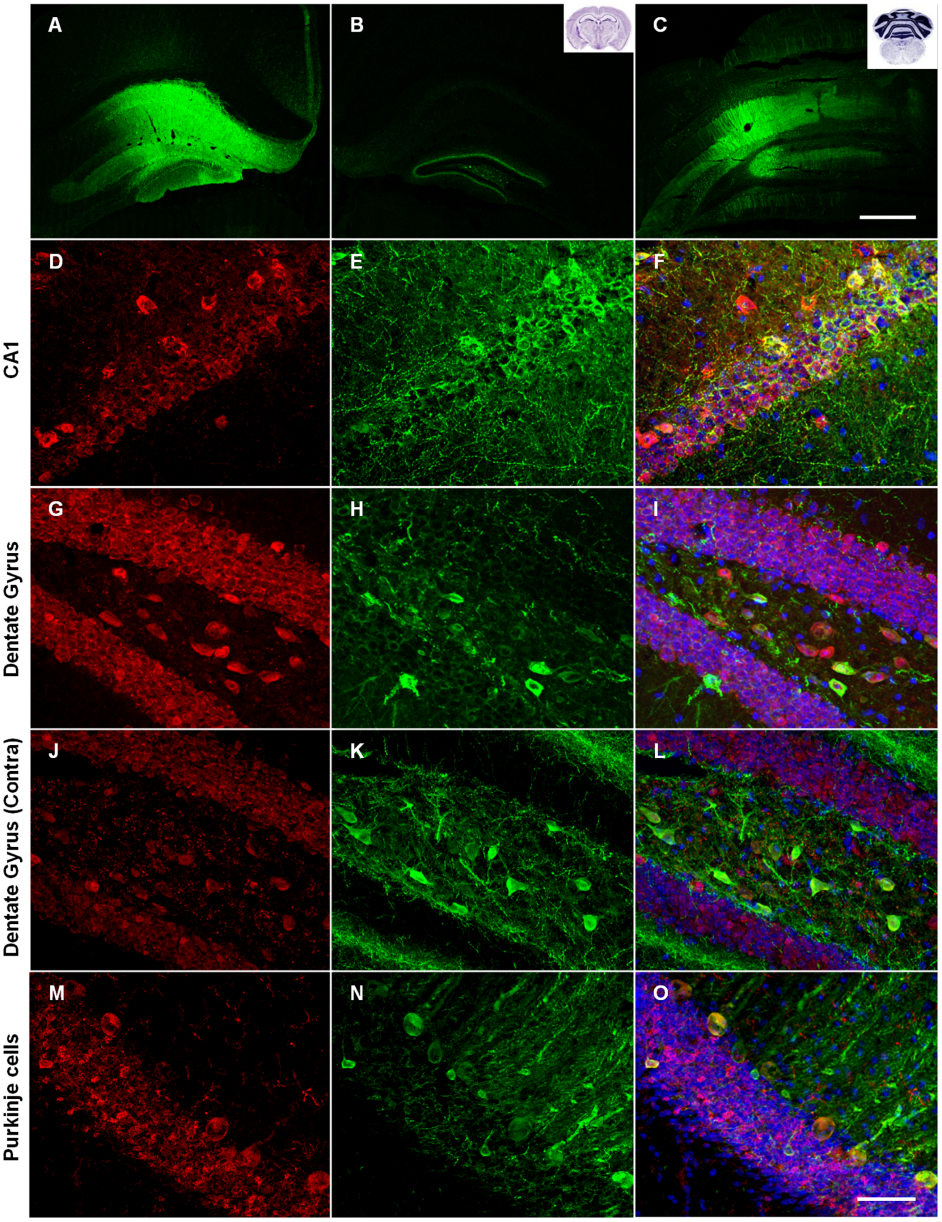
Transduction in the hippocampus and cerebellum as indicated by GFP expression. Low magnification images show widespread GFP protein throughout the hippocampus (**A**) and cerebellar cortex (**C**). GFP protein was also observed in the contralateral hippocampus (**B**). Double immunostaining for GFP (green) and NeuN (red) revealed that neurons were specifically transduced (**D–O**). Merged images (**F, I, L, O**) also show Hoechst counterstaining. Neurons in many hippocampal sub-regions were transduced including Amon’s horn (**D–F**) and dentate gyrus (**G–I**). GFP was observed in Purkinje cells and other neurons in the cerebellum (**M–O**). Scale bar  = 500 µm (**A–C**) and 50 µm (**D–O**).

rAAV2-C100-GFP, rAAV2-C100^V717F^-GFP, rAAV2-Aβ40-GFP and rAAV2-Aβ42-GFP vectors were injected into the hippocampus and cerebellum of 8 week old wild-type male mice. Transduction and resulting pathology was examined at 3 and 6 months post-injection. Injection of all AAV2 vectors resulted in wide-spread transduction in both brain regions, as shown by strong GFP expression (Figure 2A–C). Transduction efficiency appeared similar at 3 and 6 months post-injection.

Hippocampal injection resulted in transduction of all major hippocampal sub-regions including the dentate gyrus (Figure 2G–I), CA regions of Amon’s horn (Figure 2D–F) and the subiculum. There was also evidence of anterograde and retrograde transport of rAAV2 and/or GFP as a result of hippocampal injection, as shown by GFP expression in brain regions distant from the injection site including the entorhinal cortex, contralateral hippocampus (Figure 2J–L) and the anterior cingulate area of the cerebral cortex. Hippocampal rAAV2 injection resulted in transduction across a distance of up to 2.5 mm in the rostro-caudal plane.

Injection into the cerebellar cortex predominantly resulted in transduction of Purkinje cells (Figure 2M–O), while transduction was also observed in the granule cell layer to a lesser extent. There was also evidence of anterograde and retrograde transport of rAAV2 and/or GFP with GFP expression observed in the deep cerebellar nuclei, the granule cell layer of the contralateral cerebellar cortex and in the external cuneate nucleus in the medulla. Cerebellar AAV2 injection resulted in transduction across a distance of up to 1.75 mm in the rostral-caudal plane.

All AAV2 vectors specifically transduced neurons in both the hippocampus and cerebellum, which was determined by morphology and by co-localisation with neuronal markers NeuN (for examples see Figure 2F, I, L, O) and MAP2 (data not shown). Furthermore, there was no co-localisation of GFP with GFAP or IBA-1, markers of astrocytes and microglia respectively, indicating that glia were not transduced by rAAV2 vectors (data not shown), a finding supported by previous studies [17].

**Figure 3:**
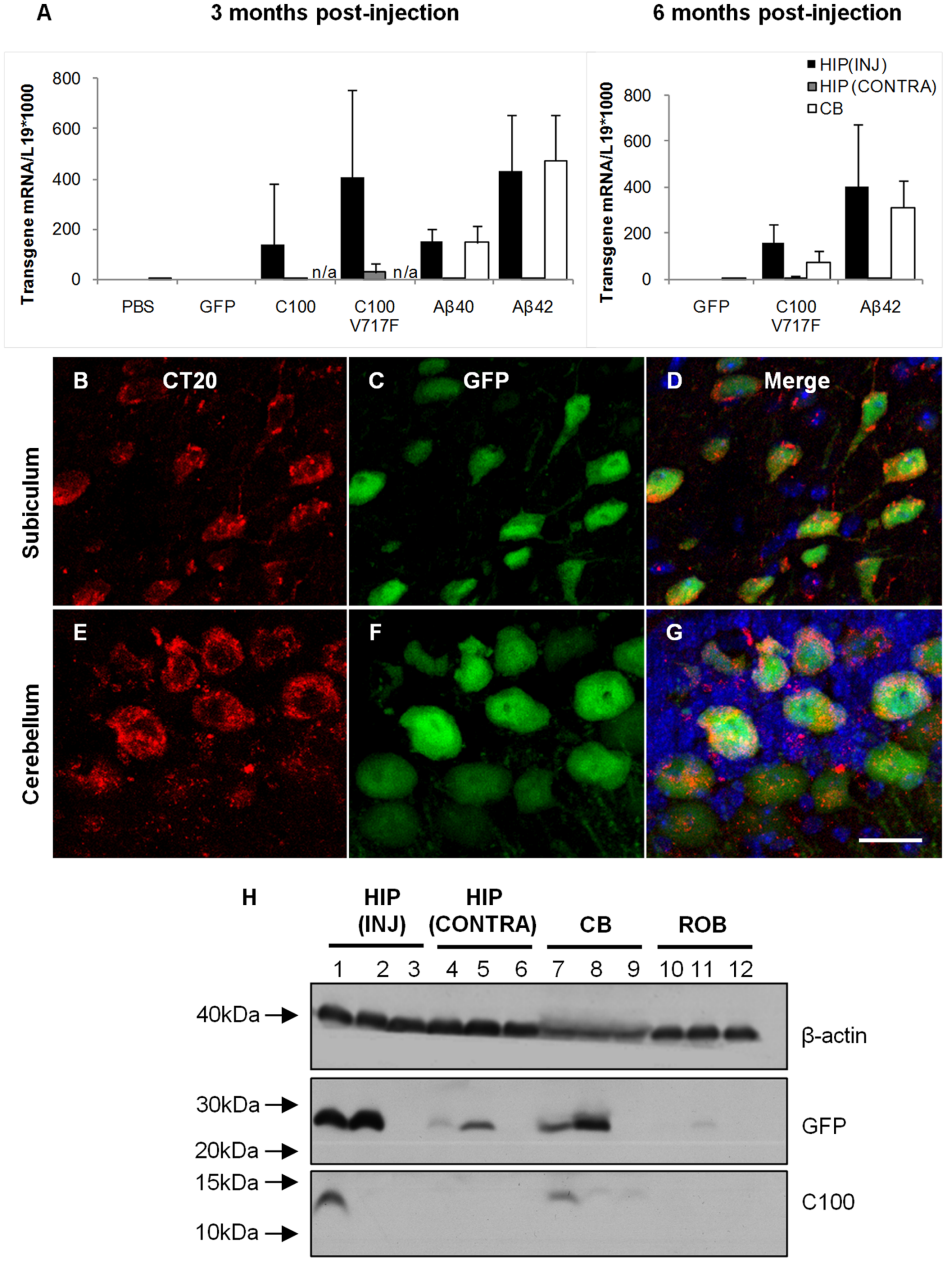
Aβ and C100 mRNA and protein expression in the hippocampus and cerebellum. mRNA for Aβ and C100 transgenes was detected using PCR (**A**). Immunostaining for C100 using CT20 antibody revealed C100 protein in GFP positive neurons (**B–G**). C100 protein was also detected using western blot (**H**). HIP (INJ): injected hippocampus, HIP (CONTRA) contralateral hippocampus, CB: cerebellum, ROB: rest of the brain. Lanes 1, 4, 7, 10; representative brain injected with AAV2-C100^V717F^-GFP, lanes 2, 5, 8, 11; representative brain injected with AAV2-Aβ42-GFP, lanes 3, 6, 9, 12; representative brain injected with AAV2-GFP.

To ensure that transduced brain regions also expressed the transgenes for the desired APP fragments in addition to GFP, qPCR was used to quantify C100-GFP, C100^V717F^-GFP, Aβ40-GFP and Aβ42-GFP transgene mRNA levels in the injected hippocampus, contralateral hippocampus and the injected cerebellar hemisphere. RNA was extracted from fixed cryosections adjacent to those used for immunohistochemistry and GFP localisation. Strong mRNA expression of the human APP fragments was observed in transduced regions of the hippocampus and cerebellum at 3 and 6 months post-injection in all mice injected with C100 and Aβ transgenes (Figure 3A). There was also lower, but detectable expression of C100 and Aβ mRNA in the contralateral hippocampus, providing evidence for retrograde transport of rAAV2 vectors and not just GFP protein. This finding supports previous studies that have shown neurons can transport AAV particles by retrograde axonal transport and that AAV then has the capability to express transgenes in distal brain regions [18], [19]. There was qualitatively similar transgene mRNA expression in the hippocampus and cerebellum, indicating a similar level of transduction in both brain regions. Importantly, no human Aβ or C100 mRNA expression was observed in any brain region of mice injected with rAAV2-GFP or PBS.

Human C100 and Aβ protein expression was also examined using Western blot and immunohistochemistry. C100 immuno-positive staining was observed throughout the cytoplasm and in primary processes of transduced neurons in the hippocampus and cerebellum (Figure 3B–G). C100 protein was also detected using Western blot in hippocampal and cerebellar tissue injected with rAAV2-C100^V717F^-GFP (Figure 3H). In contrast, Aβ could not be detected in either transduced brain region using immunohistochemistry trialling multiple antibodies against Aβ or Western blot after injection with any rAAV2 vector (data not shown). These results led to the hypothesis that Aβ may be present at very low levels and hence undetectable using Western blot or immunohistochemistry. Therefore, the high sensitivity INNO-BIA plasma Aβ forms test was used to measure Aβ40 and Aβ42 levels in the injected hippocampus and cerebellum. Aβ42 was detected in cerebellar tissue from every animal injected with rAAV2-C100^V717F^-GFP, with Aβ42 levels on average 5.9±4.3 (± standard deviation) fold higher than baseline levels. Aβ42 protein was also present in the cerebellum of one of the four animals injected with rAAV2-Aβ42-GFP, this animal having Aβ42 levels that were 3.3 fold higher than baseline levels. Aβ42 protein was also detected in the hippocampus from animals injected with either rAAV2-C100^V717F^-GFP or rAAV2-Aβ42-GFP but Aβ42 protein levels did not reach the two standard deviation criterion relative to baseline. It is important to note that, because the same viral vectors were injected into each brain region, the lack of definitive detection of Aβ42 in the hippocampus suggests that Aβ42 may be processed differently in each brain region.

Significant levels of Aβ40 were not found in the cerebellum or hippocampus after injection of either rAAV2-C100^V717F^-GFP or rAAV2-Aβ42-GFP, although a relatively high level of Aβ40 protein expression was seen in hippocampal tissue from one rAAV2-C100^V717F^-GFP injected animal. The inter-animal variation in the amount of Aβ detected, and the differences in Aβ levels between the cerebellum and hippocampus, leads to speculation that Aβ was produced but was then degraded or cleared after production, resulting in overall low levels available for detection in the murine brain samples.

### Microgliosis

**Figure 4:**
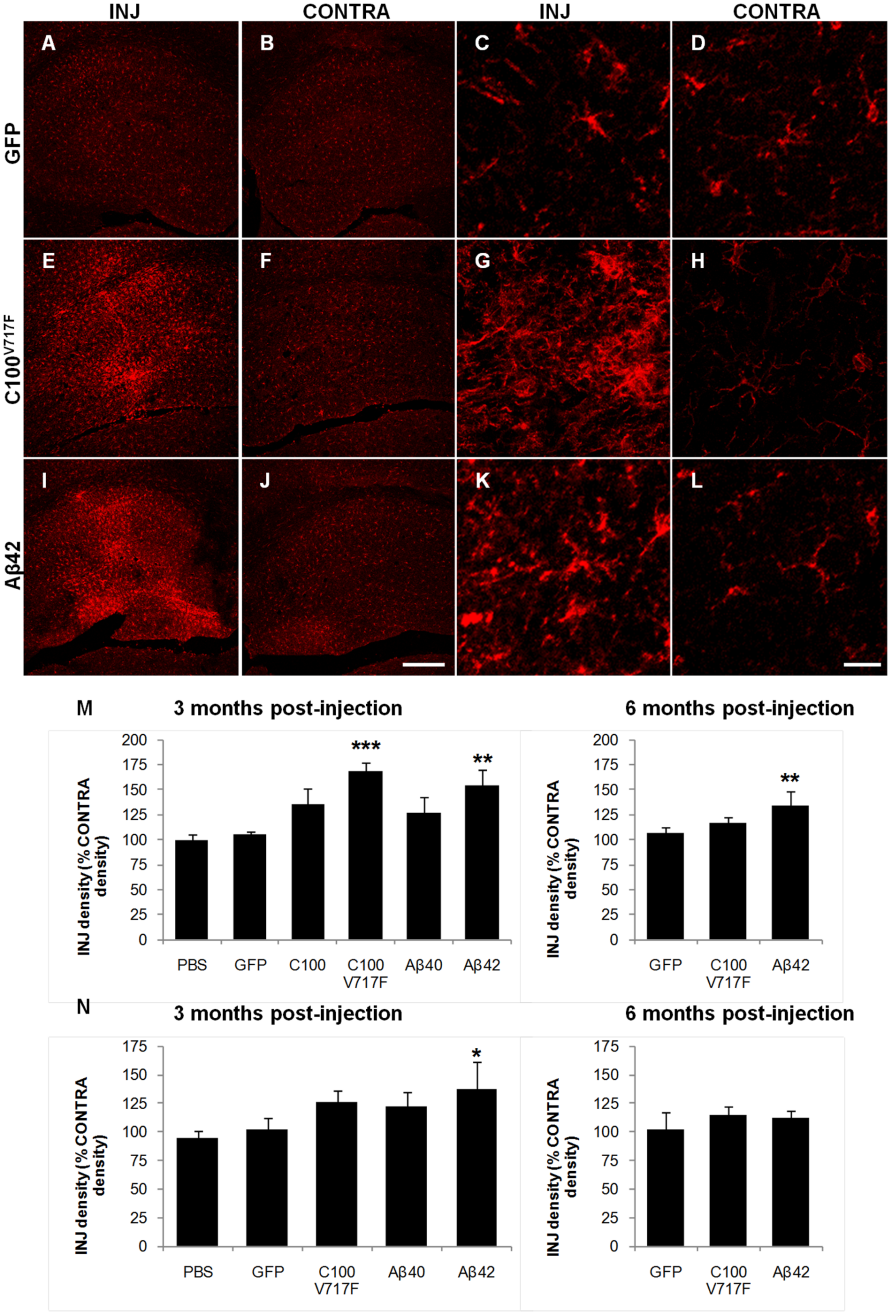
Immunostaining of microglia using IBA-1 antibody. Representative figures show microglial staining at low (**A–B, E–F, I–J**) and high magnification (**C–D, G–H, K–L**) in injected (INJ) and contralateral (CONTRA) hippocampi at 3 months post-injection with AAV2-GFP (**A–D**), AAV2-C100^V717F^-GFP (**E–H**) and AAV2-Aβ42-GFP (**I–L**). IBA-1 staining density was quantified in the hippocampus (**M**) and cerebellum (**N**) at 3 and 6 months post-injection. Data shown as density of injected region as a percentage of the corresponding contralateral region and is presented as mean ± standard deviation. ***p<0.001, **p<0.01, *p<0.05. Scale bar  = 300 µm (**A–B, E–F, I–J**) and 20 µm (**C–D, G–H, K–L**).

Immuno-positive staining density of the microglial marker IBA-1 was analysed to determine the extent of microgliosis following injection of rAAV2 vectors. This analysis technique was designed to examine wide-spread microgliosis across the whole transduced region. IBA-1 positive microglia in areas of gliosis showed morphological signs of activation including enlarged cell bodies, ramified processes and increased IBA-1 staining within individual microglia (Figure 4G, K).

In the hippocampus, injection of rAAV2-C100^V717F^-GFP and rAAV2-Aβ42-GFP resulted in significantly higher IBA-1 staining density at 3 months post-injection than that observed after rAAV2-GFP injection (Bonferroni; p<0.001 and p<0.01 respectively), while there was no significant difference in IBA-1 staining density between hippocampi injected with rAAV2-C100-GFP or rAAV2-Aβ40-GFP and rAAV2-GFP (Figure 4A, E, I, M). Microgliosis remained significantly higher in the hippocampus at 6 months post-injection with rAAV2-Aβ42-GFP than after injection of rAAV2-GFP (Bonferroni; p<0.01, Figure 4M).

In the cerebellum, injection of rAAV2-Aβ42-GFP also resulted in a significantly greater level of microgliosis at 3 months post-injection than after injection of rAAV2-GFP (Bonferroni; p<0.05; Figure 4N). Microgliosis was no longer elevated at 6 months post-injection. Therefore, combined results from the hippocampus and cerebellum suggest that microgliosis was strongly associated with Aβ42 rather than Aβ40 expression, as it was most extensive after injection with rAAV2-Aβ42-GFP and rAAV2-C100^V717F^-GFP.

### Astrogliosis and Neuronal Density

Aβ expression has been associated with astrogliosis and neuronal death, therefore the extent of astrogliosis and neuronal density were examined using immunohistochemistry for GFAP and MAP2 respectively. Quantification of GFAP staining density showed that while there was some evidence of increased astrocyte activation surrounding the injection site in both brain regions following injection of rAAV2-C100-GFP, rAAV2-C100^V717F^-GFP, rAAV2-Aβ40-GFP and rAAV2-Aβ42-GFP in comparison to after injection of rAAV2-GFP or PBS, this increase was not extensive enough to be significantly different at either 3 or 6 months post-injection (data not shown). Quantification of MAP2 staining density showed that neuronal density was not altered in the hippocampus or cerebellum as a result of injection of any rAAV2 vector at either 3 or 6 months post-injection (data not shown).

### Permeability of the Blood Brain Barrier

An unexpected consequence of injection with rAAV2 vectors was the presence of increased mouse IgG staining around the injection site, first detected after immunohistochemistry using anti-mouse secondary antibodies. Further testing showed that the increased IgG staining was observed when sections were incubated only with anti-mouse secondary antibodies, was localised to transduced brain regions, and was not observed after injection with PBS, therefore showing that it was not a technical artefact of the immunohistochemistry procedure.

**Figure 5:**
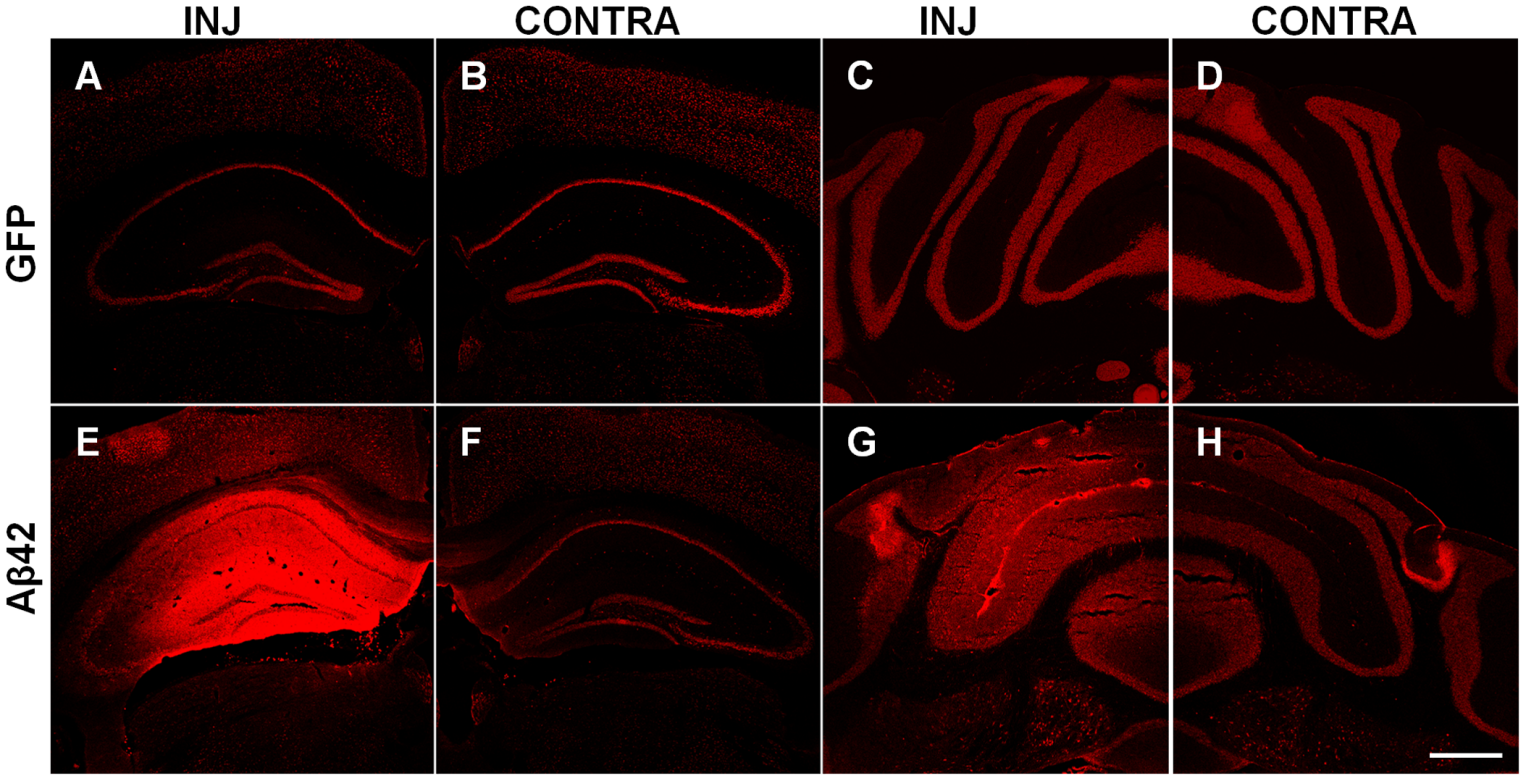
Increased mouse IgG staining in the hippocampus and cerebellum. Example images show normal IgG staining following immunohistochemistry for NeuN after AAV2-GFP injection (**A–D**) and intense IgG staining after injection of AAV2-Aβ42-GFP (**E, G**). Increased IgG staining was only observed surrounding the injection site and not in the contralateral hemisphere (**F, H**). INJ; injected region, CONTRA; contralateral region. Scale bar  = 500 µm.

Increased IgG staining was observed in the hippocampus and cerebellum after injection of rAAV2 vectors expressing APP fragments, but not after injection of rAAV2-GFP or PBS (Figure 5). The relative extracellular IgG staining intensity was graded in the hippocampus and cerebellum after immunohistochemistry for NeuN, using the IgG staining intensity of the surrounding densely packed neuronal layers as the staining intensity comparison. IgG staining intensity in the hippocampus at 3 months post-injection was significantly higher after injection of rAAV2-C100-GFP (Kruskal-Wallis; p = 0.009), rAAV2-C100^V717F^-GFP (Kruskal-Wallis; p = 0.001) and rAAV2-Aβ42-GFP (Kruskal-Wallis; p = 0.016) than after injection of AAV2-GFP. There was also a non-significant trend for increased IgG staining in the hippocampus after injection of rAAV2-Aβ40-GFP than after injection of AAV2-GFP at 3 months post-injection (Kruskal-Wallis; p = 0.056). Similarly, IgG staining intensity in the cerebellum was significantly higher at 3 months post-injection with rAAV2-C100^V717F^-GFP (Kruskal-Wallis; p = 0.014), rAAV2-Aβ40-GFP (Kruskal-Wallis; p = 0.014) and rAAV2-Aβ42-GFP (Kruskal-Wallis; p = 0.013) than after injection with rAAV2-GFP. In contrast, IgG staining intensity was no longer significantly elevated at 6 months post-injection with any rAAV2 vector in either the hippocampus or cerebellum, indicating that the increased IgG in the brain decreased with time.

**Figure 6:**
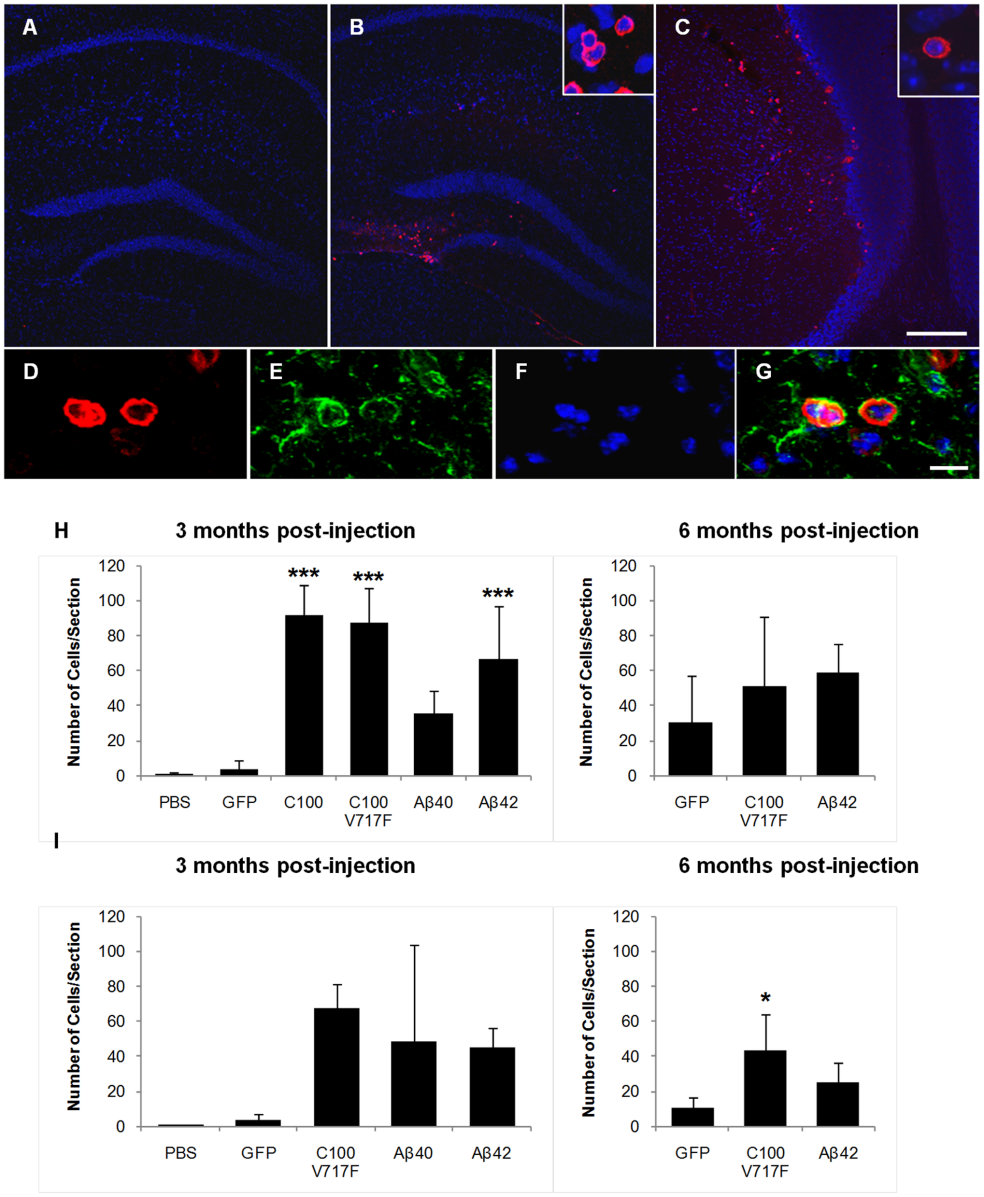
IgG positive cells in the hippocampus and cerebellum. Mouse IgG positive cells (red) were frequently observed in the hippocampus (**B**) and cerebellum (**C**) after injection with AAV2-Aβ40-GFP, AAV2-Aβ42-GFP, AAV2-C100-GFP and AAV2-C100^V717F^-GFP in comparison to after AAV2-GFP injection (**A**). Sections were counterstained with Hoechst (blue). Inserts show distinctive morphology of IgG positive cells. High magnification images of mouse IgG positive cells co-labelled with IBA-1 are shown in **D–G** (**D**: IgG staining, **E**: IBA-1 staining, **F**: Hoechst, **G**: merged image). The total number of IgG positive cells was counted in 3 sections per brain in both the hippocampus (**H**) and cerebellum (**I**) in the injected hemisphere at 3 and 6 months post-injection. Scale bar  = 200 µm (**A–C**) and 10 µm (**D–G**). INJ: injected hemisphere, CONTRA: contralateral hemisphere, ***p<0.001, *p<0.05.

Staining for mouse IgG also strongly labelled small, round cells that were predominantly observed in transduced regions of the hippocampus and cerebellum (Figure 6-A–C). Immunohistochemistry revealed that these IgG positive cells were also immunopositive for IBA-1 (Figure 6D–G), a calcium binding peptide produced specifically by monocytes and activated microglia [20]. The number of IgG positive cells was counted and averaged across three serial sections in the hippocampus and cerebellum. The vast majority of IgG positive cells were observed throughout the injected hippocampus and cerebellum and in the lateral ventricles adjacent to the injected hippocampus, with a small number of cells also observed in the contralateral hemispheres in both brain regions. Almost no IgG positive cells were seen after control PBS injections. 3 months post-injection, there were significantly more IgG positive cells in the hippocampus after injection with rAAV2-C100-GFP, rAAV2-C100^V717F^-GFP and rAAV2-Aβ42-GFP than after injection with rAAV2-GFP (Figure 6H; Bonferroni; p<0.001). In comparison, while the number of IgG positive cells in hippocampi at 3 months post-injection with rAAV2-Aβ40-GFP was higher than after rAAV2-GFP injection, this difference was not statistically significant. There was no longer a significant difference between groups in the number of IgG positive cells in the hippocampus at 6 months post-injection (Figure 6H).

The number of IgG positive cells was also increased in the cerebellum after injection with rAAV2 vectors containing APP fragments, but high inter-animal variation meant that these increases were not statistically significant at 3 months post-injection (Bonferroni; Figure 6I). However, analysis using the less stringent LSD post-hoc test found that injection with rAAV2-C100^V717F^-GFP resulted in significantly more infiltrating cells than injection with rAAV2-GFP (LSD; p = 0.049). This effect was still observed at 6 months post-injection (Bonferroni; p<0.05).

### Gene Expression

PS1, apoE, clusterin and synaptophysin gene expression was examined in the injected hippocampus, contralateral hippocampus and injected cerebellar hemisphere at 3 and 6 months post-injection. ApoE and clusterin expression was also examined to determine if expression of genes involved in Aβ clearance was altered [21]. Synaptophysin is a synaptic protein marker and was therefore examined as an additional marker of neurodegeneration and PS1 expression was examined to determine if C100 mediated endogenous PS1 expression, hence altering production of Aβ. Altered levels of each of these genes and the resulting proteins have also been previously associated with AD [22], [23], [24], [25]. Differences in gene expression between groups were only deemed significant if the statistical difference was consistently observed after normalisation to two housekeeping genes; L19 and PPIA. It was found that there was not a significant difference in expression of any gene examined as a result of injection of any vector at 3 or 6 months post-injection in either the hippocampus or cerebellum.

## Discussion

Injection of rAAV2 vectors expressing transgenes for human Aβ40, Aβ42, C100 and C100^V717F^ into the mouse hippocampus and cerebellum resulted in wide-spread transduction in both brain regions and the development of some pathological changes characteristic of AD, most notably increased microgliosis and increased permeability of the blood brain barrier.

Injection of rAAV2-Aβ42-GFP and rAAV2-C100^V717F^-GFP in the hippocampus resulted in significantly increased IBA-1 density at 3 months post-injection, while injection of rAAV2-Aβ40-GFP and rAAV2-C100-GFP did not, suggesting that Aβ42 may be a more potent mediator of microgliosis than Aβ40. It is known that Aβ attracts and causes activation of microglia [26], [27], but it has been previously suggested that this may only occur in response to fibrillar Aβ [1], [28], [29], [30]. The association between fibrillar Aβ and microgliosis is further supported by the fact that the onset of gliosis in AD transgenic mice is closely linked to the onset of plaque deposition [31], [32], and manipulating the amount of plaque deposition results in similar changes in the extent of microgliosis [33], [34], [35]. However, the results from this study suggest that microgliosis can also occur in response to soluble forms of Aβ, as no plaques were observed after injection of any rAAV2 vector.

The rAAV2 vectors used in this study also resulted in pathology indicative of increased permeability of the blood brain barrier, which was most extensive in the hippocampus at 3 months post-injection. Injection of rAAV2 vectors into the hippocampus resulted in increased brain IgG and increased numbers of IgG/IBA-1 positive cells. IgG cannot cross the blood brain barrier and is only found in the brain under pathological conditions [36]. As a result, IgG is a well established marker of blood brain barrier permeabilisation and has been shown to be a good alternative to other markers of blood brain barrier disruption such as Evans blue dye staining [37], [38], [39], [40], [41]. Cells with similar morphology to the IgG positive cells observed in this study have been characterised previously and a large number of these cells is also a common marker of blood brain barrier disruption [42]. Previous studies have suggested that these cells are leukocytes and as the cells observed in this study were also immuno-positive for IBA-1, this suggests that they were leukocytes of monocyte-macrophage lineage [43], in agreement with previous studies [37], [44].

In the hippocampus at 3 months post-injection, blood brain barrier disruption was more extensive after exposure to Aβ42, via expression of Aβ42 directly or the C100 and C100^V717F^ precursors. In comparison, while IgG staining intensity and numbers of infiltrating cells after injection with rAAV2-Aβ40-GFP were elevated, these changes were not significantly different from that observed after injection with rAAV2-GFP. Blood brain barrier disruption is a pathological feature of AD [45], [46] and previous studies have hypothesised that it may be directly caused by Aβ [42], [47], [48]. The results from this study not only support the hypothesis that Aβ expression may directly cause blood brain barrier disruption, but also suggest that Aβ42 may be a more potent mediator of blood brain barrier disruption than Aβ40.

Injection of rAAV2 vectors did not induce widespread astrogliosis or altered neuronal density in either the hippocampus or cerebellum. Activation of astrocytes in response to Aβ expression was observed to some extent, however it was primarily localised to the injection site, in contrast to the more extensive microgliosis. Previous studies have found Aβ to cause activation and migration of astrocytes [49], [50], [51]. However, it has also been shown that the activation of astrocytes in AD is dependent upon the conformation and aggregation state of Aβ; astrocytes surrounding dense core plaques become activated, while astrocytes surrounding diffuse plaques or those not associated with aggregated Aβ do not show signs of activation and can often show signs of atrophy [52], [53]. Therefore, it is possible that the lack of extensive astrogliosis observed in this study was due to the lack of aggregated, fibrillar Aβ following injection of viral vectors. The lack of widespread neurodegeneration observed in this study is consistent with previous studies that have shown that Aβ is not a potent mediator of neurodegeneration *in vivo* [54]. It is now becoming more accepted that tau hyperphosphorylation and the development of neurofibrillary tangles is more likely to be a mediator of neurodegeneration in AD than Aβ [55], [56], [57]. It is interesting that synaptophysin mRNA levels were unaffected as Aβ, particularly soluble oligomers of Aβ, has been shown to decrease expression of synaptic markers, including synaptophysin [32], [58], [59], [60], [61]. However, this finding is not consistent across all studies [62], [63], indicating that decreased synaptophysin expression may not be a direct consequence of Aβ expression and that other additional factors may be necessary. It is important to note that only gross neurodegeneration would have been observed by the quantification measures used in this study and that the use other neuronal and cell death markers may have provided a more specific indication of neurodegeneration.

Western blot and immunohistochemical processing failed to detect significant Aβ40 and Aβ42 protein expression in transduced brain regions. We do not believe that this was due to the use of inefficient viral vectors because Aβ42 was detected in tissues injected with rAAV2-C100^V717F^-GFP or rAAV2-Aβ42-GFP using the more sensitive ELISA technique, thus proving that these vectors were capable of producing Aβ. Furthermore, there was strong transduction of all bi-*cis*tronic rAAV2 vectors in the hippocampus and cerebellum, as shown by high expression levels of transgene mRNA and post-IRES GFP protein, the latter produced only after C100 or Aβ protein translation. Finally, injection of rAAV2-Aβ40-GFP and rAAV2-Aβ42-GFP resulted in the development of obvious brain pathology that was unique to each Aβ isoform and was not present after injection of vehicle or rAAV2-GFP controls, strongly suggesting that the Aβ produced as a result of rAAV2 vector injection was responsible for the observed pathologies. We hypothesise that the inability to detect Aβ using the less sensitive immunohistochemistry and western blotting methods, and the observation of variance between animals in the amount of Aβ detected using ELISA, was due to rapid clearance and/or degradation of Aβ. Aβ levels are highly regulated *in vivo* by rapid clearance across the blood brain barrier, phagocytosis by glia and degradation by multiple enzymes. Enhancement of any of these mechanisms could result in low Aβ levels. Indeed, some of the pathology observed *in vivo* including microgliosis and infiltration of cells from the periphery indicate that Aβ degradation may have been increased in response to expression of Aβ as both microglia and infiltrating monocytes are capable of Aβ phagocytosis and enhance degradation [44], [64], [65], [66], [67], [68]. Increased Aβ clearance may also explain the lack of Aβ40 detected using the ELISA-mutiplex assay, as this isoform is more readily cleared and degraded than Aβ42 [69], [70]. Furthermore, the Indiana mutation promotes the preferential production of Aβ42 rather than Aβ40, therefore the presence of this mutation and the hypothesised increased clearance of Aβ could account for the very low levels of Aβ40 observed after injection of rAAV2-C100^V717F^-GFP. Lack of available tissue prevented ELISA-based quantification of Aβ40 levels after injection of rAAV2-Aβ40-GFP.

An intriguing finding was that, while significant levels of Aβ42 protein were detected in the cerebellum but not in the hippocampus, injection of rAAV2 vectors into the hippocampus resulted in greater pathological changes than those seen in the cerebellum. Why there was this apparent paradox of increased Aβ42 but reduced pathology in the cerebellum is not clear, but these observations do suggest that there are differences in the way different brain regions process and respond to C100 and/or Aβ. Previous studies have tried to determine why the cerebellum is less vulnerable to AD pathology. It has been shown that the cerebellum contains all of the necessary proteins to produce Aβ [71], [72], and that plaques do eventually appear in the cerebellum as AD pathology advances [73], indicating that the cerebellum is capable of producing amyloid pathology. Nonetheless, the cerebellum consistently has fewer plaques and lower levels of insoluble Aβ and intracellular Aβ42 [73], [74], [75] than other brain regions that are primarily affected in AD such as the hippocampus and cortex. It seems that the cerebellum is better equipped to prevent AD pathology from progressing. A recent study reported that secreted metabolites produced from cerebellar neurons reversed AD brain pathology in AD transgenic mice, while metabolites from hippocampal neurons exacerbated pathology [76]. The exact proteins or pathways involved in the protection of the cerebellum in AD are not yet known, but it has been suggested that this may be specifically due to enhanced clearance or degradation of Aβ [77], [78]. The present data suggest an alternative hypothesis, that cerebellar cells may be intrinsically less responsive to the presence of Aβ and/or C100. Future research is needed to further examine why AD brain pathology develops differently in different brain regions as this could help determine what initiates the development of AD brain pathology.

Vectors expressing the C100 transgene were more effective at consistently producing higher amounts of Aβ than vectors directly expressing Aβ transgenes, both *in vitro* and *in vivo*. This most likely resulted from the more physiological method of production of Aβ from C100, in comparison to the non-physiological production by direct expression of either Aβ40 or Aβ42. Direct expression of Aβ may not be optimal for Aβ accumulation, possibly due to Aβ production occurring in the incorrect sub-cellular location. Previous *in vitro* studies have shown that fusing Aβ and C100 to a signal protein that directs expression in the secretory pathway greatly increases the amount of Aβ detected after plasmid transfection [79], [80], hence suggesting that sub-cellular location of Aβ may be important for expression.

A further aim of this study was to determine if the effects of transduction with rAAV2 vectors expressing APP fragments were exacerbated at 6 months post-injection in comparison to 3 months post-injection. This was not found to be the case in either brain region as less extensive pathological changes were observed at 6 months post-injection. The level of transduction was similar at 3 and 6 months post-injection, therefore the less extensive pathology observed at 6 months post-injection is unlikely to be a result of any technical issues associated with long-term transduction. Instead, it is possible that brain regions may have adapted to the long-term expression of C100 and/or Aβ and as a result became better equipped to deal with the consequent pathology, such as by increasing levels of Aβ degrading enzymes or increasing anti-inflammatory proteins. However, further studies are necessary to confirm this hypothesis.

In conclusion, the use of viral vectors to over-express Aβ and C100 is a promising technique with which to examine the consequences of Aβ expression in mature CNS tissues *in vivo*. Results from this study provide evidence that Aβ42 causes greater pathology than Aβ40, particularly by promoting microgliosis and inducing abnormal permeability changes in the blood brain barrier.
